# Neck and Wrist Circumferences as Indicators of Metabolic Alterations in the Pediatric Population: A Scoping Review

**DOI:** 10.3390/children8040297

**Published:** 2021-04-13

**Authors:** Evelyn Valencia-Sosa, Guillermo Julián González-Pérez, Erika Martinez-Lopez, Roberto Rodriguez-Echevarria

**Affiliations:** 1 Departamento de Salud Pública, Centro Universitario de Ciencias de la Salud (CUCS), Universidad de Guadalajara, Sierra Mojada 950, Guadalajara, Jalisco 44340, Mexico; envs_621@hotmail.com (E.V.-S.); ggonzal56@gmail.com (G.J.G.-P.); 2 Instituto de Nutrigenética y Nutrigenómica Traslacional, Departamento de Biología Molecular y Genómica, Centro Universitario de Ciencias de la Salud (CUCS), Universidad de Guadalajara, Sierra Mojada 950, Guadalajara, Jalisco 44340, Mexico; erikamtz27@yahoo.com.mx

**Keywords:** metabolic alterations, children obesity, scoping review, neck circumference, wrist circumference

## Abstract

Neck circumference (NC) and wrist circumference (WrC) have been proposed as practical and inexpensive tools with the capacity to indicate metabolic alterations to some extent. Nevertheless, their application in the pediatric population is relatively recent. Thus, the aim of this scoping review was to review and analyze the reported evidence regarding the correlation of NC and WrC with metabolic alterations in the pediatric stage. The literature search was performed in January 2021 in seven indexes and databases. A total of 26 articles published between 2011 and 2020 were included. Most significant results were grouped into three categories: serum lipid profile, glucose homeostasis, and blood pressure. The parameter that showed the most significant results regardless of the anthropometric indicator analyzed for association was blood pressure. In contrast, total cholesterol and LDL-cholesterol showed non-significant associations along with conflicting results. We conclude that the use of NC and WrC, in addition to other well-established indicators, could facilitate the identification of metabolic alterations, specifically in plasma insulin and blood pressure. In fact, further studies are required to address the potential use of NC and WrC as predictors of early metabolic alterations, especially in countries with a fast-growing prevalence in obesity.

## 1. Introduction

Anthropometric measurements are practical tools applied in the clinical nutrition area. One of the main objectives is to estimate body composition. In the pediatric population, these tools are particularly convenient to assess growth and development [1]. Remarkably, body mass index (BMI) remains as the most widely used indicator by health care practitioners despite the limited information it provides about body fat proportion and distribution. In this regard, a large number of reports over the last two decades have pointed out that body fat is a major factor for the development of chronic diseases [2,3,4]. In fact, central adiposity appears to have a more detrimental impact on metabolic homeostasis when compared to general adiposity [5].

Currently, there exist several advanced methodologies to assess body fat distribution, such as computed axial tomography (CAT) and magnetic resonance imaging (MRI). Nevertheless, these techniques are unaffordable for most of the population due to the high cost they represent; consequently, they remain to be widely used for research purposes [6]. For this reason, it is important to study and propose novel, reliable, low-cost, and less invasive anthropometric measurements to be applied as indicators in both clinical practice and large epidemiological due to the fast-growing prevalence of child obesity worldwide [7].

During the last decade, neck circumference (NC) and wrist circumference (WrC) have been recently proposed as practical and inexpensive tools with the capacity to indicate elevated central adiposity and insulin resistance in children and adolescents [8,9,10,11,12,13,14]. Furthermore, there exists evidence of anatomical and physiological explanations why these measurements might be strongly associated with metabolic alterations. In the case of NC, it mainly illustrates the accumulation of fat depots in the cervical area as a result of significant weight gain overtime. As has been widely acknowledged, an excessive upper body fat accumulation holds a strong relationship with metabolic alterations [4,15]. As for WrC, this measurement targets bone width that is expected to increase during childhood and puberty. In fact, bone mass can be regulated by the actions of insulin during growth. In this regard, it is believed that, under insulin resistance conditions, when insulin-targeted tissues do not response properly to this hormone, bone formation might be promoted instead [16,17]. By contrast, some controversial data have been reported in the sense that metabolic syndrome might be associated with an increased risk for diminished bone density in adolescents and adults. [18,19].

Noteworthy, the application of NC and WrC in the pediatric population is relatively recent, especially WrC. Additionally, it is important to remark that, since different measuring techniques have been used, the evidence for the usefulness and value of these measurements in clinical and epidemiological scenarios appear to be insufficient. In this regard, a scoping review on the literature might bring some clarity on the relevance of these measurements. Thus, the aim of this study is to review and analyze the reported evidence regarding the correlation of NC and WrC with metabolic alterations in the pediatric population.

## 2. Materials and Methods

This scoping review was conducted in accordance with the Arksey and O’Malley [20] framework. This scoping review addressed the following question: What biomarkers for assessing metabolic alterations have been significantly associated with NC and WrC? To answer this question, only obesity-associated metabolic disorders were considered (serum lipid profile, glucose homeostasis, and blood pressure).

### 2.1. Literature Search Strategy

The literature search was performed in January 2021 in the following indexes and databases: PubMed, ScienceDirect, Wiley Online Library, Scopus, Springer Link, Web of Science, and Scielo. The following word combinations were used: (“neck circumference” OR “wrist circumference”) AND (“metabolic risk” OR “metabolic alterations” OR “blood pressure” OR “lipid profile” OR “blood sugar”) AND (“children” OR “pediatric population”). The search was first conducted in the indexes and then in the databases. Keywords, boolean operators, and the use of special characters were adapted accordingly (Supplementary Materials Table S1).

### 2.2. Selection Criteria and Data Extraction

Studies were screened and evaluated by two reviewers. In order to evaluate the relevance of the articles retrieved, specific selection criteria were applied for each section (Table 1).

Generally, the title was first evaluated, followed by the abstract. Both sections had to meet the established criteria. If this was the case, the full-text was downloaded and saved in Mendeley-Reference Management Software. If an abstract did not show enough criteria to be immediately selected, then the full-text file was downloaded or requested by e-mail from the corresponding author of the article for further evaluation. Importantly, no restriction for year publication was applied.

The selected storage tool for the retrieved material was Microsoft OneDrive. Three additional backup files were created on Google Drive, Dropbox, and regular e-mail inbox. Then, a file on Microsoft Excel was created containing several named sheets according to the specific searches and retrievals (indexes and databases). Within every Excel sheet, information such as total results, selected items, date, and time of retrieval was registered along with the entered keywords (Supplementary Materials Table S1). Finally, all full-text articles were stored in an electronic folder tagged by author’s last name and year of publication.

### 2.3. Study Selection and Data Analyisis

**Figure 1:**
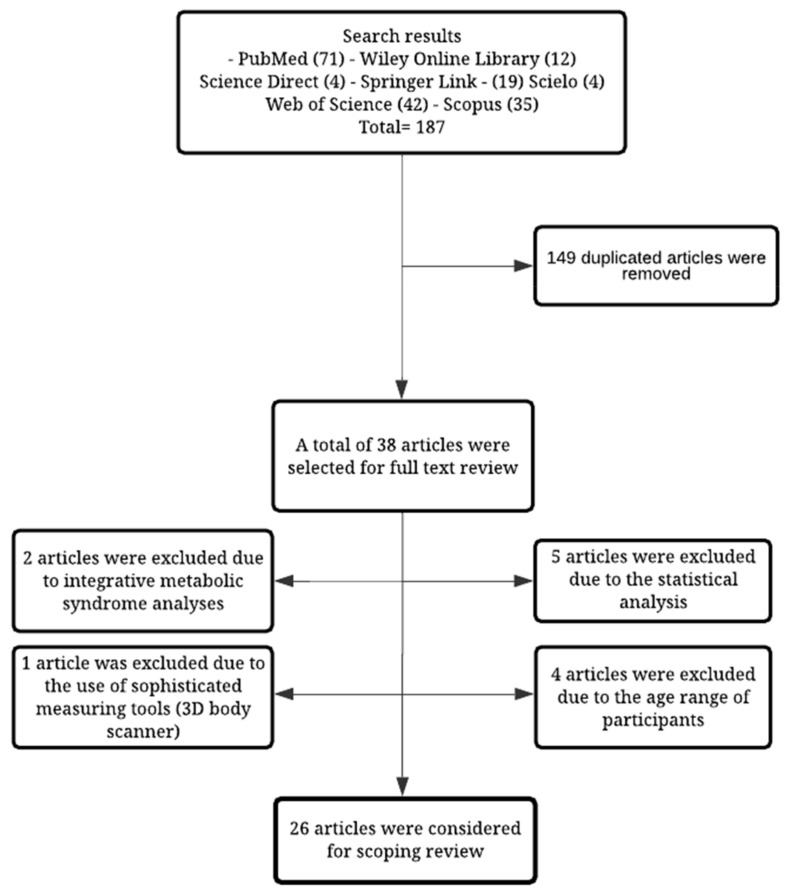
Flow chart of the literature search process.

A total of 187 articles were retrieved in the first search. From these, 149 duplicated texts were eliminated. Additionally, five articles were excluded because their analyses did not use either correlation coefficients, bivariate regression models, or multivariate regression models for variable evaluation. Furthermore, four articles were excluded due to the age range of the enrolled participants, two articles were not included due to merging data analyses based on metabolic syndrome classification, and one more article was excluded due to the use of sophisticated tools for assessing body composition (3D scanner). In summary, 26 articles were included for this scoping review (Figure 1) that were published between 2011 and 2020.

For data synthesis, a literature review matrix was created. The most relevant components for classification were first author’s last name and first name, year of publication, country, anthropometric measurement, methodology, sample size, age of participants, statistical analysis, and significant outcomes. Data were independently compiled by two reviewers and results were approved by a third researcher.

### 2.4. Methological Quality Assessment

To assess the methodological quality of the studies, we used the Newcastle–Ottawa quality assessment scale based on a star-rating system [21,22]. This was performed independently by two authors. This scale, when applied to cross-sectional studies, includes three main sections: selection, comparability, and outcome, where the maximum score was ten stars. Furthermore, we included one case-control and one cohort study, which were evaluated based on selection, comparability, and exposure/outcome. In this case, the maximum score was nine stars. Rates were classified as 10–9: very good, 8–7: good, 6–5: satisfactory, and ≤5: unsatisfactory (Table 2).

## 3. Results

### 3.1. General Characteristics

Most of the included articles carried out a cross-sectional design, except for one case-control [45] and one prospective study [46]. According to the geographical region, seven of the studies were set out in Europe [7,14,24,27,29,32,45], eight in America [25,26,33,35,39,40,41,42], eight in Asia [28,30,31,34,38,43,44,46], and three in Africa [23,36,37]. Based on the anthropometric indicator, 19 articles approached NC, seven articles WrC, and two studied both anthropometric indicators [28,31]. Furthermore, 14 articles recruited subjects at public schools [7,23,26,28,29,30,31,32,33,38,40,42,44,45] and 12 articles at outpatient clinics [14,24,25,27,34,35,36,37,39,41,43,46]. Most studies included schoolchildren and adolescents with age ranges between 5 and 18 years, except for one study that included 3-year-old preschool children [32].

Regarding the methodological quality of the included studies, we assigned the following classification: two studies as “very good”, 14 studies as “good”, eight studies as “satisfactory”, and two studies as “unsatisfactory” (Table 2). Furthermore, data are presented as the magnitude and direction of the associations along with their statistical significance. A summary of the data extracted from all articles is shown in Table 3.

### 3.2. Neck Circumference

A total of 21 articles studying NC were examined. The most common anatomical site of measurement was “at the level of the thyroid cartilage” (*n* = 15) [23,26,28,31,32,34,35,36,39,40,41,42,43,44,45], whereas the second approach was “below the thyroid cartilage” (*n* = 6) [7,25,29,30,33,37]. Furthermore, the most investigated variables to be correlated with NC were as follows: systolic blood pressure (SBP), diastolic blood pressure (DBP), fasting plasma glucose (FPG), high-density lipoprotein (HDL), and triglycerides (TG). Among these variables, serum lipid profile and glucose were measured using enzymatic colorimetric assays in most of the studies (*n* = 15), whereas the rest stated using “standard laboratory procedures” or simply no specification in this regard (*n* = 3). Also, plasma insulin (*n* = 12) was measured through ELISA kit (*n* = 4), chemiluminescence (*n* = 2), and electrochemiluminescence (*n* = 3), whereas three studies did not specify the methodology used. Finally, blood pressure was the only variable studied across all articles. The most important data extracted from this parameter were: the use of manual sphygmomanometer (*n* = 7), automatic digital monitor (*n* = 7), unspecified (*n* = 7), from which only seven articles reported performing the measurement more than once (in duplicates or triplicates).

#### 3.2.1. Serum Lipid Profile

Four studies found a positive correlation between total cholesterol (TC) and NC (*r* = 0.04 to 0.47 and R^2^ = 0.01) [35,37,39,43], however, only one study reported statistically significant results (*r* = 0.27 to 0.47, *p* < 0.05) [43]. In contrast, five articles reported negative associations between these two variables (*r* = −0.04 to −0.27) [7,31,36,37,40], but only two were statistically significant (*r* = −0.04 and −0.27, *p* < 0.05) [31,40]. Similarly, four studies found positive associations with low-density lipoprotein (LDL) (*r* = 0.01 to 0.24 and R^2^ = 0.01) [7,29,35,39]; from these, one report showed significant results (*r* = 0.24, *p* < 0.05) [29]. On the other hand, four studies found negative associations (*r* = −0.03 to −0.44) [31,36,37,40], only two of them being statistically significant (*r* = −0.03 and −0.18, *p* < 0.05) [37,40].

Regarding triglycerides (TG), 14 studies reported a positive association with NC (*r* = 0.01 to 0.41 and R^2^ = 0.02) [7,25,26,28,29,31,32,33,34,35,37,39,40,43], in which case half of these were statistically significant (*r* = 0.6 to 0.41 and *p* < 0.05) [7,26,29,32,33,39,43]. Also, negative associations were found in one study (*r* = −0.05) [36], but it was non-significant. Inversely, 12 studies found negative associations with HDL (*r* = −0.01 to −0.38 and R^2^ = 0.14) [7,25,26,31,32,34,36,37,39,40,43]; from these, nine were statistically significant (*r* = −0.06 to −0.38 and R^2^ = 0.14, *p* < 0.05) [7,26,31,32,33,34,39,40,43].

#### 3.2.2. Fasting Plasma Glucose, Plasma Insulin, and Homeostatic Model Assessment of Insulin Resistance (HOMA-IR)

Positive associations between total fasting plasma glucose FPG and NC were found in 11 studies (*r* = 0.01 to 0.32 and R^2^ = 0.04) [7,25,26,28,31,33,34,35,37,39,43], from which four studies reported statistical significance (*r* = 0.12 to 0.32 *p* < 0.05) [26,33,34,43]. In contrast, one article reported a non-significant and negative association between these two variables (*r* = −0.28) [36]. Regarding plasma insulin, 10 studies found positive associations (*r* = 0.12 to 0.61 and R^2^ = 0.15 [7,25,33,34,35,36,37,39,40,43], and eight of them were statistically significant (*r* = 0.19 to 0.61 and R^2^ = 0.15, *p* < 0.05) [7,25,33,34,35,39,40,43]. Similarly, 12 studies found positive associations with HOMA-IR (*r* = 0.07 to 0.62 and R^2^ = 0.13) [7,25,29,32,33,34,35,36,37,39,40,43], from which ten articles showed statistical significance (*r* = 0.07 to 0.62 and R^2^ = 0.13, *p* < 0.05) [7,25,29,32,33,34,35,39,40,43].

#### 3.2.3. Blood Pressure

Blood pressure was approached across all the included articles studying NC (*n* = 18). Only positive associations were found in the case of SBP (*r* = 0.03 to 0.93 and R^2^ = 0.34), where 17 of them were statistically significant (*r* = 0.05 to 0.93 and R^2^ = 0.34, *p* < 0.05) [7,25,26,29,30,31,32,33,34,35,39,40,41,42,43,44,45]. Interestingly, DBP was found to be positively associated with NC in 17 studies (*r* = 0.04 to 0.84 and R^2^ = 0.19), showing significant results in 16 of them (*r* = 0.04 to 0.84 and R^2^ = 0.19, *p* < 0.05) [26,29,30,31,33,34,35,36,37,39,40,41,42,43,44,45]. Finally, one study found a non-significant and negative association with DBP (*r* = −0.02) [32]. Noteworthy, one study carried out a linear regression analysis showing statistically significant results for both SBP (β = 0.61 CI 0.54 to 0.68, *p* < 0.05) and DBP (β = 1.25 CI 0.96 to 1.54, *p* < 0.05) [23].

#### 3.2.4. Liver Enzymes and Adipokines

Some articles explored the correlations between NC and liver enzymes. In this regard, alanine aminotransferase (ALT), aspartate aminotransferase (AST), and gamma-glutamyl transpeptidase (GGT) were positively associated in three articles (ALT *r* = 0.14 to 0.30; AST *r* = 0.07 to 0.32; GGT 0.2 to 0.43) [25,34,39]. However, these results were not consistent since one article reported these outcomes as significant only in boys but not in girls [34] and another one showed statistical significance in ALT (*r* = 0.25, *p* < 0.05) but not in AST or GGT [25].

Moreover, one study determined serum leptin and adiponectin levels and found statistically significant associations, positive for the former (girls *r* = 0.43 and boys *r* = 0.30, *p* < 0.05) and negative for the latter (girls *r* = −0.20 and boys *r* = −0.23, *p* < 0.05).

### 3.3. Wrist Circumference

Regarding WrC, seven articles were reviewed, in which three different anatomical sites were identified: site 1: “over the Lister tubercle of the distal radius and over the distal ulna” (*n* = 4) [14,24,27,28], site 2: “distal to the prominences of the radial and ulnar bones” (*n* = 2) [31,46], and site 3:, “at the most prominent aspect of the radial styloid process” (*n* = 1) [38]. Moreover, the most investigated variables to be correlated with WrC were SBP, DBP, FPG, HDL-C and TG. Specifically, serum lipid profile and glucose were measured using enzymatic colorimetric assays in most of the studies (*n* = 6), and one study did not report this information. In addition, plasma insulin (*n* = 3) was measured through radioimmunoassay (*n* = 2), and one article did not specify the methodology used. Finally, blood pressure was approached in six articles. The most relevant information in this regard was the use of automatic digital monitors in all studies, from which only two articles reported performing the measurement in duplicates.

#### 3.3.1. Serum Lipid Profile

One study found negative associations between WrC and serum cholesterol (TC *r* = −0.03 and LDL-C *r* = −0.02) [28]. However, these findings were non-significant. Noteworthy, no positive associations were reported between these variables. Regarding HDL-C, two articles showed significant and negative associations (*r* = −0.06 and *r* = −0–25, *p* < 0.05) [24,31], and one study found a non-significant positive association (*r* = 0.02) [28]. Furthermore, three studies reported positive associations between triglycerides (TG) and WrC (*r* = 0.01 to 0.2) [24,28,31], but only one study showed statistical significance (*r* = 0.2, *p* < 0.05) [24]. Finally, another study approached TG using a multiple regression analysis, showing a significant result (β = 0.22, *p* < 0.05) [14].

#### 3.3.2. Fasting Plasma Glucose, Plasma Insulin, and HOMA-IR

Three studies found positive correlations between WrC and FPG (*r* = 0.01 to 0.16) [24,28,31], but only one reported a statistical significance (*r* = 0.16, *p* < 0.05) [24]. Regarding plasma insulin and HOMA-IR, one study showed significant and positive correlations with this circumference (insulin *r* = 0.27 and HOMA-IR *r* = 0.28, *p* < 0.05) [24]. Finally, another study approached insulin and HOMA-IR by using a multiple regression analysis, which showed statistically significant results (β = 0.34 and β = 0.35 respectively, *p* < 0.05) [14].

#### 3.3.3. Blood Pressure

Blood pressure was approached in five articles studying WrC. In this regard, significative positive correlations were found for SBP (*r* = 0.25 to 0.52, *p* < 0.05) [24,31,38,46]. Also, one study carried out a multivariate regression analysis showing statistically significant results for SBP (girls β = 2.9 CI 1.44 to 4.37 and boys β = 2.6 CI 1.35 to 2.85, *p* < 0.05) [27]. As for DBP, significant positive correlations were shown in those four reports as well (*r* = 0.16 to 0.37, *p* < 0.05) [24,31,38,46]. Notwithstanding, one study showed non-significant outcomes for DBP in a multivariate regression analysis [27]. 

#### 3.3.4. Adipokines

One article reported a significant negative correlation between WrC and the serum adiponectin/leptin ratio (*r* = −0.37) [24].

## 4. Discussion

In this scoping review, we provide a comprehensive analysis about two anthropometric measurements, NC and WrC, as indicators for metabolic alterations in the pediatric population. The key points that we focused on for the data analysis were country, location of recruitment, measurement technique, determination of biomarkers, and significance of outcomes. Overall, most of the articles analyzed herein shared one common aim: to investigate the potential of NC and WrC as alternative anthropometric tools for identifying metabolic alterations in children and adolescents. In fact, over a half of the included studies were carried out in Europe and in the Americas. This is worth mentioning because both geographical regions encompass countries with a high prevalence of overweight and obesity in pediatric population such as Italy, Greece, USA, and Mexico [47]. Thus, research on the predictive potential of rapid, low-cost tools might be of service for population screening and for the creation of public health strategies, especially in developing countries without access to more sophisticated tools for body composition.

Methodologically, NC measurement involves a few elements that need to be considered. First, this measurement was taken at two anatomical sites. Lack of standardization across all the studies represents a factor that could deviate from objective conclusions. Remarkably, correlations were consistently significant for HDL, plasma insulin, HOMA-IR, SBP, and DBP regardless of the site of measurement. In this regard, one point to consider for further research on NC is the pubertal stage classification of the participants since this was not evaluated in all the included articles. The main reason for this is the fact that pubertal development is not only related to body fat distribution and metabolism, but also for the projection of the thyroid cartilage in males, which alters the measurement itself. In fact, only 10 of the included studies [7,14,25,27,29,33,34,39,43,48] stated using the Tanner classification in their methodology and data analyses. Furthermore, one study performed the measurement of NC “below Adam’s apple” only when this anatomical site was prominent; in other words, this study conducted both techniques and reported significant correlations for HDL, plasma insulin, HOMA-IR, SBP, and DBP [40].

On the other hand, WrC measurement was conducted differently in three sites of across the studies. Indeed, this might lead to different outcomes due to the involvement of two bones when conducting the measurement. It is noteworthy that for WrC, being a perimeter considerably shorter than NC, it would be reasonable to think that differences at a millimetric scale as a result of a different techniques could have a greater impact. However, it is important to mention that, because of the limited number of studies approaching WrC (*n* = 7), it is a difficult task to draw solid conclusions based on the site of measurement. Furthermore, some studies appeared to focus on blood pressure rather than glucose or lipid homeostasis. In this regard, consistent results were found in SBP and DBP, as well as in plasma insulin and HOMA-IR to some extent.

As for NC and its correlations with TC and LDL, the evidence seems to be non-conclusive for two reasons. First, these parameters were measured in less than 10 studies, and second, they were non-significant and consistently opposite in most cases since the direction of their magnitude were positive as well as negative (both significant and non-significant). As a matter of fact, the explanation for these negative associations were undiscussed in these articles.

By contrast, TG and HDL showed a different panorama in the articles studying NC. Remarkably, most articles studying these two parameters together [7,26,32,33,39,43] demonstrated significant correlations. Importantly, these findings are of great interest given that elevated TG and low HDL levels hold a strong clinical relevance as they have been acknowledged as risk factors for the development of cardiovascular disease in adulthood [49]. As for WrC, two studies showed significant associations in both TG and HDL together [14,24].

The analysis of glucose homeostasis comprised three components: fasting plasma glucose, insulin, and HOMA-IR. Noteworthy, about one third of the articles studying NC (four out of eleven) and one third of those studying WrC (one out of three) reported a significant and positive correlation with fasting glucose. Notably, although hyperglycemia reflects glucose intolerance, it does not provide a full dynamic perspective of insulin resistance. In this regard, plasma insulin is usually elevated (due to compensatory hyperinsulinemia) under low-grade chronic inflammation conditions [50]. As hypothesized, insulin showed a positive significant correlation with NC in most articles approaching these variables (eight out of ten). Consequently, HOMA-IR presented similar correlation results with NC given that it includes blood insulin concentration in its equation. As for WrC, the only two articles studying these correlations presented significant results. Thus, this evidence suggests the close relationship between the expansion of specific anatomical sites and adipose tissue dysfunction in the pediatric population.

Blood pressure was evidently the most studied biomarker. In fact, both SBP and DBP were statistically significant in nearly all articles studying NC [7,25,26,29,30,31,32,33,34,37,39,40,43,44,45] and WrC [24,27,31,38]. Interestingly, one of the included articles was a case-control study performed in Lithuanian adolescents. They found that subjects with an NC above the 90th percentile had greater risk for hypertension (OR  =  4.05 (3.03–5.41)) in presence of overweight/obesity and central adiposity [45]. A plausible explanation for this could be the pathophysiological link between hyperinsulinemia and hypertension, which is believed to be related to the prolonged actions of insulin on the sympathetic nervous system, although the mechanistic process has not been fully elucidated [51]. However, it is widely acknowledged that insulin resistance and hypertension frequently coexist [52]. Interestingly, other hypotheses have pointed out that hypertension derives from multiple simultaneous alterations, namely, low-grade chronic inflammation, unpaired adipokine secretion, or oxidative stress, all promoting endothelial dysfunction and vascular damage [53]. These findings support the importance of developing novel and complementary tools for chronic disease prevention. Nevertheless, we believe that the high correlations found in some studies (NC: SBP *r* = 0.93, DBP *r* = 0.84 and WrC SBP *r* = 0.52, DBP *r* = 0.37) [30,38] with blood pressure require a cautious interpretation, considering that the aim of most correlation studies is to eventually determine a predictive potential. This is mainly because blood pressure is a highly dynamic parameter orchestrated by complex and strictly regulated physiological processes. Thus, it is unlikely to predict its value using solely a body circumference. Hence, the optimal approach for the interpretation of these results could be to guide clinicians into a practical methodology for blood pressure monitoring and early identification of obesity-related hypertension.

Furthermore, the location of recruitment for all the studied subjects was considered worth discussing. Briefly, most studies could be classified into two categories: Schools (private and public) and outpatient clinics (specialized in obesity or endocrinology). Undoubtedly, these two approaches delivered an important selection bias. On the one hand, schools appeared to be the most convenient site for recruitment, either for research purposes or as part of a larger public health program. It allows researchers to massively screen a cohort at strategic points within a country or a region; notwithstanding, in such cases, it is difficult to control the optimal conditions for measuring biochemical variables in all subjects. Also, in countries with a certain degree of ethnic diversity, the outcome may be influenced by genetic-related differences unless these aspects are considered for the statistical analysis; however, this was not the case in the included studies. On the other hand, since prevalence of obesity in all the involved countries is unequal, recruiting at obesity clinics might impact the achievable sample size. This is important to consider because the correlation strength could be sensitive to sample size in certain cases. This situation can be illustrated by comparing two of the articles we discuss here. For instance, we could examine a large cohort comprising over 15,000 subjects recruited at schools that showed low correlation values (HOMA-IR *r* = 0.11 and SBP *r* = 0.05, *p* < 0.05) [32], as opposed to other study that recruited just above 100 subjects at outpatient clinics (HOMA-IR *r* = 0.44 and SBP *r* = 0.54, *p* < 0.05) [25], yet there is a plausible physiological explanation for the relationship between insulin resistance and blood pressure as we have previously mentioned.

Finally, it is important to discuss the relevance of these results in comparison to waist circumference (WC), as it has been the most widely studied measurement regarding metabolic alterations due to its relationship with central adiposity. WC is still used by health care practitioners, especially in activities such as population screening and clinical assessment. Notwithstanding, WC has some methodological issues, namely: the lack of international standardization due to ethnic-related differences and some practical obstacles, such as the need to remove clothing, which might be complicated for some individuals, especially during the winter season. WC is also significantly altered by the postprandial state and respiratory movements. Thus, it would be preferable to use alternative measurements in some cases. In fact, eleven of the included studies assessed either NC or WrC along with WC [7,26,28,29,33,34,35,41,45,46]. For instance, NC correlation values were similar in the study conducted by Hatipoglu et al. [34] in plasma insulin (NC *r* = 0.52, *p* < 0.05 versus WC *r* = 0.49, *p* < 0.05), HDL (NC *r* = −0.38, *p* < 0.05 versus WC *r* = 0.41, *p* < 0.05), and SBP (NC *r* = 0.34, *p* < 0.05 versus WC *r* = 0.32, *p* < 0.05). A similar situation can be illustrated in the article of Kalantari et al. [46] where SBP (WrC *r* = 0.38, *p* < 0.05 versus WC *r* = 0.37, *p* < 0.05) and DBP (WrC *r* = 0.18, *p* < 0.05 versus WC *r* = 0.22, *p* < 0.05) showed similar values when compared to WC. The evidence they present suggests that these two measurements can similarly identify metabolic alterations, but NC and WrC have the advantage of being more practical and less invasive than WC.

### 4.1. Strengths and Limitations

This scoping review reflects a comprehensive literature search, quality assessment, data extraction, and analysis of the differences and similarities among 26 articles conducting research on NC and WrC and their correlation with metabolic alteration parameters. We have also pointed out several areas of opportunity that should be taken into consideration for future studies.

As for the limitations of this study, we did not perform an in-depth comparison of their statistical analyses, nor have we delved into the factors involved in the possible lack of statistical power of the included studies. Furthermore, results from studies using different sites of measurement were not compared, mainly because the number of the included reports is limited, especially in the case of WrC. Finally, this work admitted only peer-reviewed articles; consequently, “grey” literature studies were omitted as a result of our design.

### 4.2. Future Directions 

The most investigated biochemical markers in these studies were HDL-C, TG, and insulin. However, two studies [24,29] determined serum adiponectin and leptin levels, showing significant correlations. Remarkably, since these molecules are found at lower concentrations compared to other chronic diseases with a more robust inflammatory component, we would recommend that future studies approach adipokines using high-sensitivity methods.

Apparently, the potential of WrC has not been sufficiently explored despite showing some interesting results. Thus, more studies approaching WrC in countries with a high prevalence of childhood obesity could bring more evidence for future meta-analyses.

## 5. Conclusions

Although the information presented here does not lead to solid conclusions to be drawn regarding the single use of NC or WrC for assessing metabolic alterations in the pediatric population, the reported evidence suggests that using these tools in addition to other well-established indicators could facilitate the identification of altered insulin sensitivity and blood pressure, especially in the case of NC. Remarkably, further studies are required to address the potential use of WrC in countries with a fast-growing prevalence in obesity.

## Figures and Tables

**Table 1:** Selection criteria.

**General**
Original articles
No restriction for study design
No restriction for year of publication
**Title**
Contains at least one of the keywords
Published in English
**Abstract**
Subjects belong to the pediatric stage (3 to 18 years of age)
Metabolic disorder indicators were assessed
**Full text**
Correlation coefficients, bivariate or multivariate regression models were used within the statistical analysis

**Table 2:** Quality assessment of the studies.

	Selection				Comparability	Outcome/Exposure *		Score	Quality
Study	Representativeness of the Sample	Sample Size	Non-Respondents	Ascertainment of the Exposure (Risk Factor)	Confounding Factors are Controlled	Assessment of the Outcome	Statistical Test		
Katamba et al. [23]	★	★	★	★★	★	★★	★	9	Very good
Luordi et al. [24]	-	-	-	★	★	★★	★	5	Satisfactory
Peña-Vélez et al. [25]	-	-	-	★	★	★★	★	5	Satisfactory
González-Cortés et al. [26]	★	★	-	★	-	★★	★	6	Satisfactory
Zampetti et al. [27]	★	★	-	★	★	★★	★	7	Good
Hanieh-Sadat et al. [28]	★	★	-	★	★	★★	★	7	Good
Castro-Piñero et al. [29]	★	★	-	★	★	★★	★	7	Good
Rajagopalan et al. [30]	★	-	-	★	-	★★	★	5	Satisfactory
Kelishadi et al. [31]	★	★	★	★	★	★★	★	8	Good
Formisano et al. [32]	★	★	★	★	-	★★	★	7	Good
Gomez-Arbelaez et al. [33]	★	★	-	★	★	★★	★	7	Good
Hatipoğlu et al. [34]	-	-	-	★	★	★★	★	5	Satisfactory
Faria et al. [35]	-	-	-	★	★	★★	★	5	Satisfactory
Abeer et al. [36]	-	-	-	★	-	★★	★	4	Unsatisfactory
Hassan et al. [37]	-	-	-	★	-	★★	★	4	Unsatisfactory
Kajale et al. [38]	★	★	-	★★	★★	★★	★	9	Very good
Da Silva et al. [39]	★	-	-	★	★★	★★	★	7	Good
Gonçalves et al. [40]	★	★	-	★	★	★★	★	7	Good
Nafiu et al. [41]	★	★	-	★	★★	★★	★	8	Good
Oliveira et al. [42]	★	-	-	★	★	★★	★	6	Satisfactory
Androutsos et al. [7]	★	-	-	★	★★	★★	★	7	Good
Kurtoglu et al. [43]	-	-	-	★	★★	★★	★	6	Satisfactory
Guo et al. [44]	★	★	★	★	★	★★	★	8	Good
Capizzi et al. [14]	★	-	-	★	★★	★★	★	7	Good
**Study**	**Representativeness**	**Case Definition**	**Selection of Controls**	**Definition of Controls**	**Comparability**	**Ascertainment and Method of Ascertainment for Cases and Controls**		**Score**	**Quality**
Kuciene et al. ^†^ [45]	★	-	★	★	★★	★★		7	Good
**Study**	**Representativeness of the exposed cohort**	**Selection of non-exposed cohort**	**Ascertainment of exposure**	**Demonstration that outcome was not present at start**	**Comparability of cohorts**	**Assessment of outcome/Length and adequacy of follow-up**		**Score**	**Quality**
Kalantari et al. ^†^ [46]	★	★	★	★	★	★★★		8	Good

* “Outcome” was evaluated in the case of cross-sectional and cohort studies, whereas “Exposure” corresponds to case-control studies. ^†^ These are studies with a maximum score of 9 stars according to the Newcastle–Ottawa quality assessment form for case-control and cohort studies. ★ Equal to one point granted in the corresponding category.

**Table 3:** Characteristics of included studies.

Author	Country	Anthropometric Indicator	Measurement Site	Subjects (Age in Years)	Statistical Analysis	Significant Outcomes
Katamba et al. [23]	Uganda	Neck circumference	At the level of the thyroid cartilage	616 (12–19)	Linear regression analysis	SBP β = 0.61 (CI 0.54–0.68), DBP β = 1.25 (CI 0.96–1.54)
Luordi et al. [24]	Italy	Wrist circumference	Over the Lister tubercle of the distal radius and over the distal ulna	280 (7–18)	Spearman correlation coefficient	HOMA−IR (*r* = 0.28), INS (0.27), ADIPOQ/LP ratio (*r* = −0.37), HDL (*r* = −0.25), TG (*r* = 0.20), FPG (*r* = 0.16), SBP (*r* = 0.40), DBP (*r* = 0.16)
Peña-Vélez et al. [25]	Mexico	Neck circumference	Around the inferior margin of the laryngeal prominence	112 (6–18)	Pearson’s correlation coefficient	SBP (*r* = 0.54), ALT (0.25), HOMA−IR (*r* = 0.42), INS (*r* = 0.44)
González-Cortés et al. [26]	Mexico	Neck circumference	At the level of the thyroid cartilage	548 (6–18)	Pearson’s correlation coefficient	Girls: HDL (*r* = −0.31), TG (*r* = 0.31), SBP (*r* = 0.33), DBP = (*r* = 0.13) Boys: HDL (*r* = −0.28), TG (*r* = 0.25), SBP (*r* = 0.27), FPG = (*r* = 0.12)
Zampetti et al. [27]	Italy	Wrist circumference	Over the Lister tubercle of the distal radius and over the distal ulna	1133 (5–16)	Multivariate regression analysis	Girls: SBP β = 2.90 (CI 1.44–4.37) Boys: SBP β = 2.60 (CI 1.35–3.85)
Hanieh-Sadat et al. [28]	Iran	Neck circumference	In the midway of the neck, between mid-cervical spine and mid anterior neck	14,138 (7–18)	Pearson’s correlation coefficient	No significant correlations
		Wrist circumference	Over the Lister tubercle of the distal radius and over the distal ulna			No significant correlations
Kalantari et al. [46]	Iran	Wrist circumference	Distal to the prominences of radial and ulnar bones.	1579 (10–19)	Pearson’s correlation coefficient	SBP (*r* = 0.38), DBP (*r* = 0.18)
Castro-Piñero et al. [29]	Spain	Neck circumference	Below the laryngeal prominence	2198 (6–18)	Pearson’s correlation coefficient	Girls: SBP (*r* = 0.42), DBP (*r* = 0.30), TG (*r* = 0.28), HOMA-IR (*r* = 0.27), LEP (*r* = 0.43), ADIPOQ (*r* = −0.20) Boys: SBP (*r* = 0.51), DBP (*r* = 0.34), TG (*r* = 0.37), HOMA-IR (*r* = 0.26), LEP (*r* = 0.30), ADIPOQ (*r* = −0.23), LDL (*r* = 0.24)
Rajagopalan et al. [30]	India	Neck circumference	Just below the laryngeal prominence and positioned perpendicular to the long axis of the neck at the level of the thyroid cartilage	500 (13–17)	Pearson’s correlation coefficient	Normal NC SBP (*r* = 0.93), DBP (*r* = 0.84) High NC SBP (*r* = 0.77), DBP (*r* = 0.54)
Kelishadi et al. [31]	Iran	Neck circumference	With the most prominent portion of the thyroid cartilage taken as a landmark	3843 (7–18)	Pearson’s correlation coefficient	Girls: SBP (*r* = 0.32), DBP (*r* = 0.26), FPG (*r* = 0.05) Boys: SBP (*r* = 0.40), DBP (*r* = 0.31), HDL (*r* = −0.08), LDL (*r* = −0.07), TC (*r* = −0.08) Total: SBP (*r* = 0.36), DBP (*r* = 0.29), TC (*r* = −0.04), HDL (*r* = −0.06), LDL (*r* = −0.03)
		Wrist circumference	Distal to the prominences of radial and ulnar bones.			Girls: SBP (*r* = 0.33), DBP (*r* = 0.25) Boys: SBP (*r* = 0.40), DBP (*r* = 0.31), FPG (*r* = − 0.01), HDL (*r* = −0.1) Total: SBP (*r* = 0.37), DBP (*r* = 0.28), HDL (*r* = −0.06)
Formisano et al. [32]	Italy Belgium Cyprus Estonia Germany Hungary Spain Sweden	Neck circumference	At the level of the thyroid cartilage	15,673 (3–10)	Partial correlation coefficient	Girls: HOMA-IR (*r* = 0.11), TG (*r* = 0.06), HDL (*r* = −0.06), SBP (*r* = 0.05) Boys: HOMA-IR (*r* = 0.07), TG (*r* = 0.06), HDL (*r* = −0.06)
Gomez-Arbelaez et al. [33]	Colombia	Neck circumference	Just below the laryngeal prominence and applied perpendicular to long axis of the neck.	669 (8–14)	Pearson’s correlation coefficient	Girls: FPG (*r* = 0.19), SBP (*r* = 0.34), DBP (*r* = 0.29), INS (*r* = 0.22), HOMA-IR (*r* = 0.23), HDL (*r* = −0.12) Boys: FPG (*r* = 0.19), SBP (*r* = 0.42), DBP (*r* = 0.31), INS (*r* = 0.25), TG (0.18), HOMA-IR (*r* = 0.27), HDL (*r* = −0.29) Total: FPG (*r* = 0.20), HDL (*r* = −0.19), TG (0.11), SBP (*r* = 0.39), DBP (*r* = 0.29), INS (*r* = 0.19), HOMA-IR (*r* = 0.21)
Hatipoğlu et al. [34]	Turkey	Neck circumference	At the level of the most prominent portion of the thyroid cartilage	248 (10–18)	Spearman correlation coefficient	Girls: INS (*r* = 0.48), HOMA-IR (*r* = 0.52), SBP (*r* = 0.34), DBP (*r* = 0.46), FPG (*r* = 0.32), HDL (*r* = −0.38) Boys: INS (*r* = 0.32), HOMA-IR (*r* = 0.33), ALT (*r* = 0.30), GGT (*r* = 0.43), AST (*r* = 0.32)
Faria et al. [35]	Brazil	Neck circumference	At the level of the thyroid cartilage	82 (10–17)	Linear regression analysis	Obese adolescents: SBP (*r*^2^ = 0.34), DBP (*r*^2^ = 0.19), HDL (*r*^2^ = 0.14), INS (*r*^2^ = 0.15), HOMA-IR (*r*^2^ = 0.13)
Abeer et al. [36]	Egypt	Neck circumference	At the level of the thyroid cartilage	50 obese children (7–12)	Pearson’s correlation coefficient	Obese: DBP (*r* = 0.28)
Hassan et al. [37]	Egypt	Neck circumference	In the midway of the neck (between midcervical spine and midanterior neck)	50 obese and 50 healthy children (7–12)	Pearson’s correlation coefficient	Obese subjects without MS: LDL (*r* = −0.44) Obese subjects with MS: DBP (*r* = 0.45)
Kuciene et al. [45]	Lithuania	Neck circumference	At the level of the thyroid cartilage	1974 (12–15)	Pearson’s correlation coefficient	Girls: SBP (*r* = 0.36), DBP (*r* = 0.15) Boys: SBP (*r* = 0.55), DBP (*r* = 0.17) Total: SBP (*r* = 0.52), DBP (*r* = 0.12)
Kajale et al. [38]	India	Wrist circumference	The most prominent aspect of the radial styloid process	6380 (6–18)	Correlation (not specified)	Girls: 6–9 yr: SBP (*r* = 0.42), DBP (*r* = 0.31); 10–14 yr: SBP (*r* = 0.41), DBP (*r* = 0.32); 15–18 yr: SBP (*r* = 0.25), DBP (*r* = 0.24) Boys: 6–12 yr: SBP (*r* = 0.52), DBP (0.37); 13–15 yr: SBP (*r* = 0.46), DBP (*r* = 0.35); 16–18 yr: SBP (*r* = 0.28), DBP (*r* = 0.24)
Da Silva et al. [39]	Brazil	Neck circumference	At the midpoint of the neck	388 (10–19)	Partial correlation coefficient	Girls Prepubertal: INS (*r* = 0.30), HOMA-IR (*r* = 0.31) Pubertal: SBP (*r* = 0.27), DBP (*r* = 0.16), INS (*r* = 0.49), HOMA-IR (*r* = 0.46), TG (*r* = 0.30), AU (*r* = 0.43), GGT (*r* = 0.27), HDL (*r* = − 0.26), ALT (*r* = 0.19) Total: SBP (*r* = 0.28), DBP (*r* = 0.18), INS (*r* = 0.43), HOMA-IR (*r* = 0.41), TG (*r* = 0.25), AU (*r* = 0.35), GGT (*r* = 0.20), HDL (*r* = −0.24), ALT (*r* = 0.17) Boys Prepubertal: SBP (*r* = 0.49), DBP (*r* = 0.43), AU (*r* = 0.54), GGT (*r* = 0.31) Pubertal: SBP (*r* = 0.45), DBP (*r* = 0.34), INS (*r* = 0.31), HOMA-IR (*r* = 0.30), LDL (*r* = 0.25), TG (*r* = 0.26), UA (*r* = 0.50), GGT (*r* = 0.36), HDL (*r* = −0.40) Total: SBP (*r* = 0.47), DBP (*r* = 0.37), INS (*r* = 0.29), HOMA-IR (*r* = 0.29), TG (*r* = 0.23), UA (*r* = 0.52), GGT (*r* = 0.34), HDL (*r* = −0.34), HbA1c (*r* = −0.17)
Gonçalves et al. [40]	Brazil	Neck circumference	At its midpoint, except when the individual had a pronounced Adam’s apple, in which case the neck circumference was measured right below it	260 (10–14)	Spearman correlation coefficient	Girls: HOMA-IR (*r* = 0.33), INS (*r* = 0.36), DBP (*r* = 0.43), SBP (*r* = 0.65), TC (−0.19), HDL (*r* = −0.26) Boys: HOMA-IR (*r* = 0.50), INS (*r* = 0.49), DBP (*r* = 0.33), SBP (*r* = 0.63), TC (−0.29), LDL (*r* = −0.26), TG (*r* = 0.20), HDL (*r* = −0.26) Total: HOMA-IR (*r* = 0.35), INS (*r* = 0.36), DBP (*r* = 0.29), SBP (*r* = 0.62), HDL (*r* = −0.27), TC (*r* = −0.27), LDL (*r* = −0.18),
Nafiu et al. [41]	USA	Neck circumference	At the level of the thyroid cartilage	1058 (6–18)	Pearson’s correlation coefficient	Girls: DBP (*r* = 0.28), SBP (*r* = 0.41) Boys: DBP (*r* = 0.23), SBP (*r* = 0.44)
Oliveira et al. [42]	Brazil	Neck circumference	At the level of the thyroid cartilage	218 (16–18)	Spearman correlation coefficient	Girls: DBP (*r* = 0.04) Boys: DBP (*r* = 0.31), SBP (*r* = 0.41)
Androutsos et al. [7]	Greece	Neck circumference	Just below the thyroid cartilage	324 (9–13)	Pearson’s and Spearman correlation coefficient	Girls: DBP (*r* = 0.20), TG (*r* = 0.22), INS (*r* = 0.35), HOMA-IR (*r* = 0.36), SBP (*r* = 0.43), HDL (*r* = −0.23) Boys: INS (*r* = 0.23), HOMA-IR (*r* = 0.23), SBP (*r* = 0.43), HDL (*r* = −0.32) Total: HDL (*r* = −0.27), SBP (*r* = 0.43), TG (*r* = 0.15), INS (*r* = 0.26), HOMA-IR (*r* = 0.26),
Kurtoglu et al. [43]	Turkey	Neck circumference	At the level of the most prominent portion of the thyroid cartilage	581 (5–18)	Spearman correlation coefficient	Prepubertal stage Girls: SBP (*r* = 0.40), DBP (*r* = 0.32), FPG (*r* = 0.21), INS (*r* = 0.42), TC (*r* = 0.27), TG (*r* = 0.21), HOMA-IR (*r* = 0.41), HDL (*r* = −0.35) Boys: SBP (*r* = 0.50), DBP (*r* = 0.34), FPG (*r* = 0.17), INS (*r* = 0.61), TC (*r* = 0.30), TG (*r* = 0.41), HOMA-IR (*r* = 0.62) Pubertal stage Girls: SBP (*r* = 0.27), DBP (*r* = 0.19), INS (*r* = 0.46), TG (*r* = 0.20), HOMA-IR (*r* = 0.45), HDL (*r* = −0.19) Boys: SBP (*r* = 0.45), DBP (*r* = 0.47), INS (*r* = 0.33), TC (*r* = 0.47), TG (*r* = 0.38), HOMA-IR (*r* = 0.34), HDL (*r* = −0.30)
Guo et al. [44]	China	Neck circumference	At the level of the thyroid cartilage	6802 (5–18)	Pearson’s correlation coefficient	Normal weight SBP (*r* = 0.45), DBP (*r* = 0.33) Overweight SBP (*r* = 0.46), DBP (*r* = 0.34) Obesity SBP (*r* = 0.48), DBP (*r* = 0.33)
Capizzi et al. [14]	Italy	Wrist circumference	Over the Lister tubercle of the distal radius and over the distal ulna	477 (mean 10.3)	Multiple regression analysis	HOMA β = 0.35, INS β = 0.34, TG β = 0.22 (CI not reported)

FPG: Fasting plasma glucose, TG: triglycerides, LDL: low density lipoprotein, TC: total cholesterol, LEP: leptin, HDL: high density lipoprotein, INS: insulin, HOMA-IR: Homeostatic model assessment of insulin resistance, SBP: Systolic blood pressure, DBP: diastolic blood pressure, ADIPOQ: adiponectin, HbA1c: glycated hemoglobin, ALT: alanine aminotransferase GGT: gamma glutamyl transferase, AST: aspartate aminotransferase, UA: uric acid, MS: metabolic syndrome, NC: neck circumference, yr: years old.

## Data Availability

Data supporting reported results are available upon request to the corresponding author.

## Supplementary Materials

The following are available online at https://www.mdpi.com/article/10.3390/children8040297/s1, Table S1: Search log.

## References

[1] Patton I., McPherson A. (2013). Anthropometric measurements in Canadian children: A scoping review: Discovery service for endeavour college of natural health library. Can. J. Blic. Heal..

[2] Coutinho C.A., Longui C.A., Monte O., Conde W., Kochi C. (2014). Measurement of neck circumference and its correlation with body composition in a sample of students in Sao Paulo, Brazil. Horm. Res. Paediatr..

[3] Martin M.L., Jensen M.D. (1991). Effects of body fat distribution on regional lipolysis in obesity. J. Clin. Invest..

[4] Vague J. (1956). The degree of masculine differentiation of obesities: A factor determining predisposition to diabetes, atherosclerosis, gout, and uric calculous disease. Am. J. Clin. Nutr..

[5] Sjöström C.D., Håkangård A.C., Lissner L., Sjöström L. (1995). Body compartment and subcutaneous adipose tissue distribution--risk factor patterns in obese subjects. Obes. Res..

[6] Ben-Noun L., Sohar E., Laor A. (2001). Neck circumference as a simple measure identifying overweight and obese patients. Obes. Res..

[7] Androutsos O., Grammatikaki E., Moschonis G., Roma-Giannikou E., Chrousos G.P., Manios Y., Kanaka-Gantenbein C. (2012). Neck circumference: A useful screening tool of cardiovascular risk in children. Pediatr. Obes..

[8] Mohebi R., Mohebi A., Sheikholeslami F., Azizi F., Hadaegh F. (2014). Wrist circumference as a novel predictor of hypertension and cardiovascular disease: Results of a decade follow up in a West Asian cohort. J. Am. Soc. Hypertens..

[9] Li Y., Liu Y., He J., Ma P., Yu L., Sun G. (2019). The association of wrist circumference with hypertension in northeastern Chinese residents in comparison with other anthropometric obesity indices. PeerJ.

[10] Luo Y., Ma X., Shen Y., Xu Y., Xiong Q., Zhang X., Xiao Y., Bao Y., Jia W. (2017). Neck circumference as an effective measure for identifying cardio-metabolic syndrome: A comparison with waist circumference. Endocrine.

[11] Yang G.-R., Yuan M.-X., Wan G., Zhang X.-L., Fu H.-J., Yuan S.-Y., Zhu L.-X., Xie R.-R., Zhang G.-D., Li Y.-L. (2019). Association Between Neck Circumference and the Occurrence of Cardiovascular Events in Type 2 Diabetes: Beijing Community Diabetes Study 20 (BCDS-20). Biomed Res. Int..

[12] Valencia-Sosa E., Chávez-Palencia C., Romero-Velarde E., Larrosa-Haro A., Vásquez-Garibay E.M., Ramos-García C.O. (2019). Neck circumference as an indicator of elevated central adiposity in children. Public Health Nutr..

[13] Maddaloni E., Cavallari I., De Pascalis M., Keenan H., Park K., Manfrini S., Buzzetti R., Patti G., Di Sciascio G., Pozzilli P. (2016). Relation of Body Circumferences to Cardiometabolic Disease in Overweight-Obese Subjects. Am. J. Cardiol..

[14] Capizzi M., Leto G., Petrone A., Zampetti S., Papa R.E., Osimani M., Spoletini M., Lenzi A., Osborn J., Mastantuono M. (2011). Wrist circumference is a clinical marker of insulin resistance in overweight and obese children and adolescents. Circulation.

[15] Lee J.J., Pedley A., Therkelsen K.E., Hoffmann U., Massaro J.M., Levy D., Long M.T. (2017). Upper Body Subcutaneous Fat Is Associated with Cardiometabolic Risk Factors. Am. J. Med..

[16] Kinjo M., Setoguchi S., Solomon D.H. (2007). Bone mineral density in adults with the metabolic syndrome: Analysis in a population-based U.S. sample. J. Clin. Endocrinol. Metab..

[17] Schwartz A.V. (2003). Diabetes Mellitus: Does it Affect Bone?. Calcif. Tissue Int..

[18] Da Silva V.N., Fiorelli L.N.M., Da Silva C.C., Kurokawa C.S., Goldberg T.B.L. (2017). Do metabolic syndrome and its components have an impact on bone mineral density in adolescents?. Nutr. Metab..

[19] Yu C.-Y., Chen F.-P., Chen L.-W., Kuo S.-F., Chien R.-N. (2017). Association between metabolic syndrome and bone fracture risk. Medicine.

[20] Arksey H., O’Malley L. (2005). Scoping studies: Towards a methodological framework. Int. J. Soc. Res. Methodol. Theory Pract..

[21] The Newcastle-Ottawa Scale (NOS) for Assessing the Quality of Nonrandomised Studies in Meta-Analyses. http://www.ohri.ca/programs/clinical_epidemiology/oxford.asp.

[22] Modesti P.A., Reboldi G., Cappuccio F.P., Agyemang C., Remuzzi G., Rapi S., Perruolo E., Parati G. (2016). Panethnic Differences in Blood Pressure in Europe: A Systematic Review and Meta-Analysis. PLoS ONE.

[23] Katamba G., Agaba D.C., Migisha R., Namaganda A., Namayanja R., Turyakira E. (2020). Prevalence of hypertension in relation to anthropometric indices among secondary adolescents in Mbarara, Southwestern Uganda. Ital. J. Pediatr..

[24] Luordi C., Maddaloni E., Bizzarri C., Pedicelli S., Zampetti S., D’Onofrio L., Moretti C., Cappa M., Buzzetti R. (2020). Wrist circumference is a biomarker of adipose tissue dysfunction and cardiovascular risk in children with obesity. J. Endocrinol. Invest..

[25] Penã-Vélez R., Garibay-Nieto N., Cal-Y-Mayor-Villalobos M., Laresgoiti-Servitje E., Pedraza-Escudero K., Garciá-Blanco M.D.C., Heredia-Nieto O.A., Villanueva-Ortega E. (2020). Association between neck circumference and non-alcoholic fatty liver disease in Mexican children and adolescents with obesity. J. Pediatr. Endocrinol. Metab..

[26] González-Cortés C.A., Téran-García M., Luevano-Contreras C., Portales-Pérez D.P., Vargas-Morales J.M., Cubillas-Tejeda A.C., Cossío-Torres P.E., Aradillas-García C. (2019). Neck Circumference and Its Association with Cardiometabolic Risk Factors in Pediatric Population. Medicina.

[27] Zampetti S., Campagna G., Lucantoni F., Marandola L., D’Onofrio L., Chiesa C., Pacifico L., Vania A., Buzzetti R., Leto G. (2018). Wrist circumference is associated with increased systolic blood pressure in children with overweight/obesity article. Hypertens. Res..

[28] Ejtahed H.-S., Qorbani M., Motlagh M.E., Angoorani P., Hasani-Ranjbar S., Ziaodini H., Taheri M., Ahadi Z., Beshtar S., Aminaee T. (2018). Association of anthropometric indices with continuous metabolic syndrome in children and adolescents: The CASPIAN-V study. Eat. Weight Disord. Stud. Anorex. Bulim. Obes..

[29] Castro-Piñero J., Delgado-Alfonso A., Gracia-Marco L., Gómez-Martínez S., Esteban-Cornejo I., Veiga O.L., Marcos A., Segura-Jiménez V. (2017). Neck circumference and clustered cardiovascular risk factors in children and adolescents: Cross-sectional study. BMJ Open.

[30] Rajagopalan A., Balaji N. (2017). Association of Neck Circumference and Obesity with Blood Pressure among Adolescents in Urban and Rural Population in North Tamil Nadu. J. Nat. Sci. Biol. Med..

[31] Kelishadi R., Heidari-Beni M., Qorbani M., Motamed-Gorji N., Motlagh M.E., Ziaodini H., Taheri M., Ahadi Z., Aminaee T., Heshmat R. (2017). Association between neck and wrist circumferences and cardiometabolic risk in children and adolescents: The CASPIAN-V study. Nutrition.

[32] Formisano A., Bammann K., Fraterman A., Hadjigeorgiou C., Herrmann D., Iacoviello L., Marild S., Moreno L.A., Nagy P., Van Den Bussche K. (2016). Efficacy of neck circumference to identify metabolic syndrome in 3–10 year-old European children: Results from IDEFICS study. Nutr. Metab. Cardiovasc. Dis..

[33] Gomez-Arbelaez D., Camacho P.A., Cohen D.D., Saavedra-Cortes S., Lopez-Lopez C., Lopez-Jaramillo P. (2016). Neck circumference as a predictor of metabolic syndrome, insulin resistance and low-grade systemic inflammation in children: The ACFIES study. BMC Pediatr..

[34] Hatipoğlu N., Doğan S., Mazıcıoğlu M.M., Kurtoğlu S. (2016). Relationship between Neck Circumference and Non-Alcoholic Fatty Liver Disease in Childhood Obesity. J. Clin. Res. Pediatr. Endocrinol..

[35] Faria B., Escrivão M., Nogueira P. (2016). Is Neck Circumference a Marker for Cardiovascular Risk in Obese Adolescents?. Int. J. Child Heal. Nutr..

[36] Atef A., Ibrahim A., Hassan N.E., Elmasry S.A., Elashry G.I. (2015). Neck circumference as a novel screening method for estimating fat distribution and metabolic complications in obese children. Egypt. Pediatr. Assoc. Gaz..

[37] Hassan N.E., Atef A., El Masry S.A., Ibrahim A., Al-Tohamy M., Rasheed E.A., Elashry G.I.A. (2015). Is neck circumference an indicator for metabolic complication of childhood obesity?. Open Access Maced. J. Med. Sci..

[38] Kajale N.A., Khadilkar A.V., Chiplonkar S.A., Khadilkar V.V. (2014). Body fat indices for identifying risk of hypertension in Indian children. Indian Pediatr..

[39] Da Silva C.C., Zambon M.P., Vasques A.C., Rodrigues A.M., Camilo D.F., Antonio M.A., Cassani R.S., Geloneze B. (2014). Neck circumference as a new anthropometric indicator for prediction of insulin resistance and components of metabolic syndrome in adolescents: Brazilian Metabolic Syndrome Study. Rev. Paul. Pediatr..

[40] Gonçalves V.S.S., Faria E.R., Franceschini S.C.C., Priore S.E. (2014). Neck circumference as predictor of excess body fat and cardiovascular risk factors in adolescents. Rev. Nutr..

[41] Nafiu O.O., Zepeda A., Curcio C., Prasad Y. (2014). Association of neck circumference and obesity status with elevated blood pressure in children. J. Hum. Hypertens..

[42] De Oliveira A.V., de Jesus Costa A.C.P., Pascoal L.M., dos Santos L.H., Chaves E.S., de Araújo M.F.M. (2014). Correlation between antrhopometric indicators and blood pressure in adolescents. Texto Context. Enferm..

[43] Kurtoglu S., Hatipoglu N., Mazicioglu M.M., Kondolot M. (2012). Neck circumference as a novel parameter to determine metabolic risk factors in obese children. Eur. J. Clin. Invest..

[44] Guo X., Li Y., Sun G., Yang Y., Zheng L., Zhang X., Sun Z., Ma H., Wang N., Jiang M. (2012). Prehypertension in Children and Adolescents: Association with Body Weight and Neck Circumference. Intern. Med..

[45] Kuciene R., Dulskiene V., Medzioniene J. (2015). Association of neck circumference and high blood pressure in children and adolescents: A case-control study. BMC Pediatr..

[46] Kalantari S., Khalili D., Asgari S., Fahimfar N., Hadaegh F., Tohidi M., Azizi F. (2017). Predictors of early adulthood hypertension during adolescence: A population-based cohort study. BMC Public Health.

[47] Ng M., Fleming T., Robinson M., Thomson B., Graetz N., Margono C., Mullany E.C., Biryukov S., Abbafati C., Abera S.F. (2014). Global, regional, and national prevalence of overweight and obesity in children and adults during 1980-2013: A systematic analysis for the Global Burden of Disease Study 2013. Lancet.

[48] Junge J., Engel C., Vogel M., Naumann S., Löffler M., Thiery J., Kratzsch J., Kiess W., Körner A. (2017). Neck circumference is similarly predicting for impairment of glucose tolerance as classic anthropometric parameters among healthy and obese children and adolescents. J. Pediatr. Endocrinol. Metab..

[49] Umer A., Kelley G.A., Cottrell L.E., Giacobbi P., Innes K.E., Lilly C.L. (2017). Childhood obesity and adult cardiovascular disease risk factors: A systematic review with meta-analysis. BMC Public Health.

[50] De Rooij S.R., Nijpels G., Nilsson P.M., Nolan J.J., Gabriel R., Bobbioni-Harsch E., Mingrone G., Dekker J.M. (2009). Low-grade chronic inflammation in the relationship between insulin sensitivity and cardiovascular disease (RISC) population: Associations with insulin resistance and cardiometabolic risk profile. Diabetes Care.

[51] Zou K.H., O’Malley A.J., Mauri L. (2007). Receiver-Operating Characteristic Analysis for Evaluating Diagnostic Tests and Predictive Models. Circulation.

[52] Lastra G., Syed S., Kurukulasuriya L.R., Manrique C., Sowers J.R. (2014). Type 2 diabetes mellitus and hypertension: An update. Endocrinol. Metab. Clin. N. Am..

[53] Wirix A.J.G., Kaspers P.J., Nauta J., Chinapaw M.J.M., Kist-van Holthe J.E. (2015). Pathophysiology of hypertension in obese children: A systematic review. Obes. Rev..

